# Self-anointing behaviour in captive titi monkeys (*Callicebus* spp.)

**DOI:** 10.5194/pb-5-1-2018

**Published:** 2018-01-11

**Authors:** João Pedro Souza-Alves, Natasha M. Albuquerque, Luana Vinhas, Thayane S. Cardoso, Raone Beltrão-Mendes, Leandro Jerusalinsky

**Affiliations:** 1 Department of Zoology, Federal University of Pernambuco, Recife, 50670-901, Brazil; 2 Post-graduate programme in Ecology and Conservation, Federal University of Sergipe, São Cristóvão, 49100-000, Brazil; 3 Graduate in Biological Sciences, Catholic University of Salvador, Salvador, 41740-090, Brazil; 4 Graduate in Biological Sciences, Federal University of Sergipe, São Cristóvão, 49100-000, Brazil; 5 National Centre of Research and Conservation of the Brazilian Primates, Chico Mendes Institute for Biodiversity Conservation, João Pessoa, 58010-480, Brazil

## Abstract

Self-anointing behaviour using *Bauhinia* sp. was reported in two captive titi monkeys (*Callicebus coimbrai* and *Callicebus barbarabrownae*). The study was carried out from October 2013 to May 2014 during an experimental study investigating the gut passage time of these individuals at the Getúlio Vargas Zoobotanical Park, north-eastern Brazil. Although leaves, petioles and flowers of *Bauhinia* contain chemical substances that could affect the presence of ectoparasites, it is unclear if titi monkeys demonstrate self-anointing behaviour as a method of self-medication. However, due to the presence of large glands in *C. coimbrai* and *C. barbarabrownae* chests, and the high frequency of occurrence observed for the adult male, we cautiously suggest that the use of *Bauhinia* may be linked to olfactory communication.

## 1 Introduction

Non-human animals have been found to self-medicate or to scent-mark most commonly through self-anointing, fur-rubbing and scent-rubbing behaviours in order to alleviate or to control illnesses (Rodriguez and Wrangham, 1993) commonly caused by leaves and/or invertebrates (i.e. zoopharmacognosy). Self-anointing behaviour occurs when a solitary and/or group of animals rub directly or chew and mix plant or insect material with saliva on their fur (Huffman, 2011). Wild and captive primates have been documented to use plants and invertebrates as medicinal agents to repel or kill ectoparasites (i.e. mosquitos, ticks) and microbial pathogens, as well as to treat wounds, and rubbing materials against individuals' bodies, aiming to optimise the coverage of medicines applied to both individuals or groups (Westergaard and Fragaszy, 1987; Baker, 1996; Valderrama et al., 2000; Weldon et al., 2003; Falótico et al., 2007; Verderane et al., 2007; Meunier et al., 2008; Morrogh-Bernard, 2008; Bowler et al., 2015).

Alternatively, self-anointing may be a form of group scent-marking behaviour, just as urine washing, faecal marking and even the use of plant extracts in some primate species (Ueno, 1991; Campbell, 2000; Leca et al., 2007; Paukner and Suomi, 2008, 2012). For instance, non-human primates such as spider (*Ateles* spp.), owl (*Aotus* spp.) and capuchin monkeys (*Cebus* and *Sapajus*) have been found to use self-anointing as a method of scent marking (olfactory communication or enhanced sociality) between individuals (Laska et al., 2007; Lynch-Alfaro et al., 2012; Jefferson et al., 2014). Given that individuals often interact with each other while self-anointing, this behaviour may reinforce social bonds and may be a form of social convention such as handclasp grooming in chimpanzees (McGrew and Tutin, 1978; Campbell, 2000; Carnegie et al., 2006; Laska et al., 2007; Leca et al., 2007; Paukner and Suomi, 2008, 2012) and hand sniffing in white-faced capuchins (Perry et al., 2003). During self-anointing behaviour, the animals may either (i) bite and chew the plant parts causing the production of saliva, possibly indicating a medicinal function; or (ii) they may only bite and squeeze the plants without the formation of saliva, demonstrating a social function (Baker, 1996; Huffman, 2011). This behaviour may occur in three ways: (i) when only one individual rubs a substance on itself to reach specific body regions (chest rubbing and muzzle rubbing); (ii) socially, when individuals rub their bodies against those of other members of the group in order to cover their whole body with the substance in question (Lynch-Alfaro et al., 2012); and (iii) when they rub substances on scent glands found on their bodies (Campbell, 2000).

Self-anointing behaviour has been reported previously in titi monkeys. For instance, *Plecturocebus discolor* and *Plecturocebus* *toppini* have been observed to use chewed *Tetrathylacium* (Salicaceae) as well as Annonaceae and Bignoniaceae plant leaves (Carrillo-Bilbao et al., 2005). Recently, an adult male of *Plecturocebus oenanthe* was reported to use *Piper aduncum* leaves (Piperaceae) for fur rubbing after chewing and squeezing the leaves (Huashuayo-Llamocca and Heymann, 2017). Additionally, *Plecturocebus moloch*The species referred by Moynihan's (1966) study are *Plecturocebus cupreus* and *Plecturocebus ornatus* according to the recent taxonomy (Byrne et al., 2016). individuals were reported to rub their chests, likely in order to spread any odoriferous secretion from skin scent glands (Moynihan, 1966). There is still limited data available on in situ and ex situ behaviours for titi monkeys, *Callicebus coimbrai* and *Callicebus barbarabrownae*. Self-anointing behaviour has been studied in capuchins due to their anecdotal behaviour. Reporting this behaviour at an individual or group level helps to increase our knowledge on the range of species within the primate order that presents such behaviour and what the possible behavioural contexts of self-anointing are for a given species. It is therefore essential to identify new behaviours in these species in order to better understand the social behaviour of poorly studied monogamous primate groups. Here, we report the use of leaves, flowers and petioles of *Bauhinia* sp. (Fabaceae) by two captive titi monkeys (*C. coimbrai* and *C. barbarabrownae*) applied during self-anointing behaviour. Our results are discussed in the light of self-medication and olfactory communication hypotheses.

## 2 Methods

**Figure 1:**
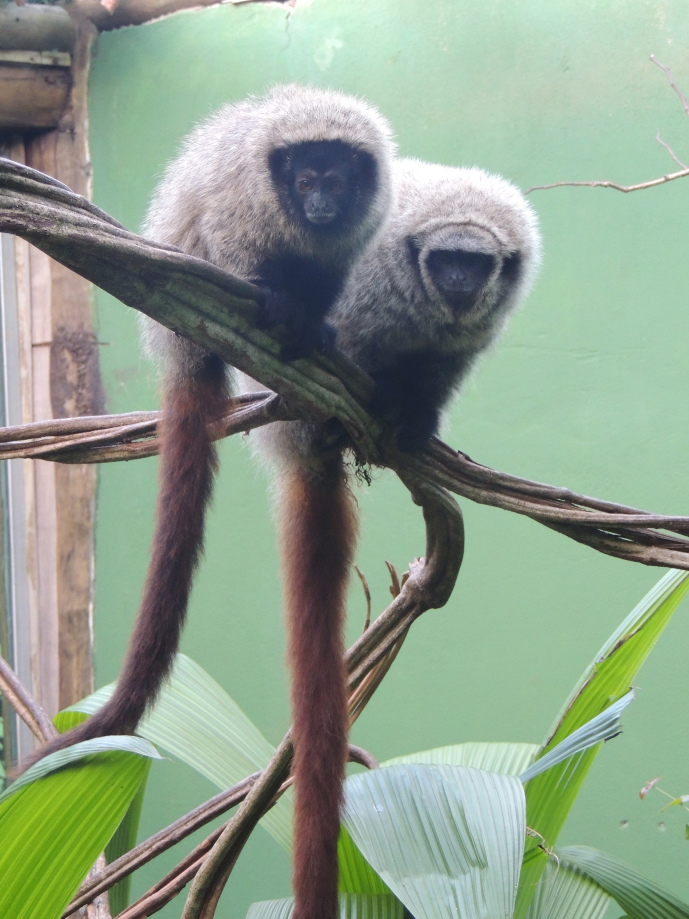
Two captive titi monkeys, *Callicebus coimbrai* (left) and *Callicebus barbarabrownae* (right), monitored during the study at the Getúlio Vargas Zoobotanical Park. Photo: João Pedro Souza-Alves.

The study, in which the observations were reported, was conducted at the Getúlio Vargas Zoobotanical Park (13${}^{\circ }$0${}^{\prime }$23${}^{\prime \prime }$ S; 38${}^{\circ }$30${}^{\prime }$20${}^{\prime \prime }$ W) in Salvador, Bahia, north-eastern Brazil. The study subjects were two captive individual titi monkeys, an adult male *Callicebus coimbrai* and an adult female *Callicebus barbarabrownae* (Fig. 1), rescued from the illegal pet trade and included in the study. The animals were monitored between October 2013 and May 2014 (excluding April 2014) for a total of 362 h (monthly average: 51 h 49 min ±05 h 16 min). Monitoring took place from dawn (05:00 h) to dusk (18:00 h), with the main goal of verifying the gut passage time of these individuals, in a specific experimental design. However, we deployed “all occurrences” sampling (Altmann, 1974) whenever scent marking did or did not follow the self-anointing behaviours that were observed. During the study period, the animals were kept in a 2m×3m×3 m enclosure. Dry trunks placed on the ground, a pot with fresh water replaced daily and two plant species were (*Eugenia uniflora*, Myrtaceae; and *Bauhinia* sp.) added to the enclosure. In addition, dry lianas were present in the enclosure, with the aim of providing environmental enrichment as well as for the welfare of the animals.

## 3 Results

Self-anointing was observed on 29 occasions. In all cases only parts of *Bauhinia* sp. were involved. Self-anointing behaviour was observed a total of 25 times for the male and 4 times for the female. The animals bit leaves (21 cases), petioles (5 cases) and flowers (3 cases) from the tree and kneaded them with either one or both hands. There were no observations of individuals rubbing the plant on each other. The two captive individuals strongly rubbed themselves with the squeezed plant material against the chest–abdominal area. This behaviour commonly involved rubbing only one item of pressed plant parts during each event on the body and lasted between 15 and 30 s, without the scent marking after such behaviour.

## 4 Discussion

The genus *Bauhinia* is widely distributed across Africa, Asia and South America. In Brazil, the genus occurs throughout the country (61 species) and across a variety of biomes (Atlantic Forest, Amazon, Caatinga, Cerrado, Pampas and Pantanal) (Vaz, 2015). Their leaves and stem–bark have been used frequently in folk medicine as a remedy for a wide variety of ailments such as diabetes, infections, pain and inflammation (Cechinel Filho, 2000; da Silva et al., 2000; da Silva and Cechinel Filho, 2002; Cavalcanti and Favoreto, 2005). The major chemical constituents of *Bauhinia* sp. are flavonoids and kaempferitrin, although additional secondary compounds are present, such as terpenes, steroids, aromatic acids, quinones, lactones, and alkaloids, among others (da Silva and Cechinel Filho, 2002; Mali et al., 2007). Only ingestion (i.e. via infusions or decoctions) of *Bauhinia* sp. extract by humans has been previously described (Pinheiro et al., 2017; Sengupta and Ahmed, 2015). In contrast, the chemical substances (anethole, apiole, carvone, cineole, dillapiole, phenylpropanoids) found in the leaves and fruits (e.g. *Citrus*, *Clematis*, *Piper*, *Sloanea*) used by *Cebus capucinus *and *P. oenanthe* during self-anointing are considered to be insecticides (Baker, 1996; Huashuayo-Llamocca and Heymann, 2017). Although the *Bauhinia* sp. used by titi monkeys has important chemical substances that may have medicinal purposes for humans and non-human primates, it is unclear if its use is related to the self-medication behaviour that occurred in the captive titis.

Neotropical primates have been recorded using olfactory cues to signal territorial, social and reproductive behaviours (Di Fiore et al., 2006; Heymann, 2006; Jefferson et al., 2014). According to Lynch-Alfaro et al. (2012), restricted locations on the body and lack of sociality for self-anointing behaviour could indicate that medicinal use is less likely to occur. It has also been suggested that captive and wild *Ateles geoffroyi* individuals use scent-marking behaviour as olfactory communication on the fur of specific body parts, for example chest-to-mouth scratching, chest rubbing, and rubbing of sternal and axillary areas over either a vertical or horizontal surface (Klein and Klein, 1971; Campbell, 2000). In contrast, *Piper* leaves were rubbed against the abdominal area of *P. oenanthe* in a possible case of self-medication (Huashuayo-Llamocca and Heymann, 2017). In this study, we observed two adult titi monkeys chest rubbing with squeezed leaves, with a higher frequency of occurrence for the adult male (n=25 events). The high frequency of occurrence observed for the adult male and the accentuated odour of the flowers and leaves may plausibly support the hypothesis of olfactory communication between the captive individuals. Moynihan (1966) described scent-marking behaviour for *P. moloch* and indicated the presence of large glands that release odoriferous secretions in the centre of an individual's chest. Similarly, an adult male individual of *C. coimbrai* was reported to rub their chest fur when in the presence of another adult male (intergroup) and a pregnant adult female (intragroup) in the wild (J. P. Souza-Alves, unpublished data). During this behaviour, the adult male did not rub any external substances on the fur i.e. the individual only rubbed the sternal gland with the hand, likely inducing scent marking. This aspect reinforces the hypothesis of olfactory communication between captive individuals. Therefore, the self-anointing behaviours reported here may not necessarily be linked to self-medication or to repelling of parasites (Baker, 1996; Morrogh-Bernard, 2008), although some chemical substances found in the plant may act as a repellent. However, we can speculate that they may be associated with olfactory communication between captive titis.

## Data Availability

No data sets were used in this article.
